# 

**Published:** 1875-03

**Authors:** 


					﻿Vol. 22.
MARCH, 1875.
No. 3.
TWENTY-SECOND YEAR.
HALL’S
JOURNAL OF HEALTH.
PUBLISHED BY THE AMERICAN AND FOREIGN
PUBLICATION CO., 137 EIGHTH ST., NEW YORK.
Agents for the Trade:
AMERICAN NEWS COMPANY, 121 NASSAU STREET.
NEW YORK NEWS COMPANY, 18 BEEKMAN STREET.
Terms, Two Dollars a Year.
SINGLE NUMBER, 20 CENTS.
				

